# Associations between general and central obesity and hypertension among children: The Childhood Obesity Study in China Mega-Cities

**DOI:** 10.1038/s41598-017-16819-y

**Published:** 2017-12-04

**Authors:** Yaling Zhao, Liang Wang, Bo Xue, Youfa Wang

**Affiliations:** 1 Global Health Institute, Xi’an Jiaotong University Health Science Center, Xi’an, Shaanxi 710061 P. R. China; 2 Department of Epidemiology and Biostatistics, School of Public Health, Xi’an Jiaotong University Health Science Center, Xi’an, Shaanxi 710061 P. R. China; 3 Department of Biostatistics and Epidemiology, College of Public Health, East Tennessee State University, Johnson City, TN 37614 USA; 4 Institute for Nutrition and Food Safety Risk Monitoring, Shaanxi Provincial Centre for Diseases Control and Prevention, Xi’an, Shaanxi 710054 P. R. China

## Abstract

In this study, we examined the associations of general and central obesity and hypertension among Chinese children. Data was collected from 1626 children aged 7–16 years and their parents in four mega-cities across China. Mixed effect models examined associations of general and central obesity with hypertension, and between body mass index (BMI), waist circumference (WC), waist-to-height ratio (WHtR), systolic blood pressure (SBP) and diastolic blood pressure (DBP). The prevalence of general obesity, central obesity, and hypertension among the children was 11.1%, 19.7%, and 9.0%, respectively. More boys had general and central obesity than girls (15.2% vs. 6.9%; 27.4% vs. 11.7%, respectively; both P < 0.0001). Sex difference in hypertension rate was not statistically significant (9.3% in boys vs. 8.8% in girls, P = 0.7341). Both SBP and DBP were positively associated with BMI, WC, and WHtR, regardless of sex and region. General obesity (OR = 5.94, 95% confidence interval [CI]: 3.69–9.55) and central obesity (OR = 3.45, 95% CI: 2.27–5.23) were strongly associated with hypertension. The prevalence of general obesity, central obesity, and hypertension was high among Chinese children in the four mega-cities across China. Children’s BMI, WC, and WHtR were positively associated with their SBP and DBP. Obese children were 3–6 times more likely to have hypertension.

## Introduction

The prevalence of overweight, obesity, and hypertension in children and adults has increased rapidly in China over the past decade. Recent national data show that among Chinese adults, the prevalence of overweight, obesity, and hypertension increased from 22.8%, 7.1%, and 18.8% in 2002 to 30.1%, 11.9%, and 25.2% in 2012, respectively^1^. Among Chinese children, the combined prevalence of overweight and obesity has increased rapidly over the past decades, from less than 3% in 1985 to 19.2% in 2010^2^. Overweight and obesity are more prevalent in urban areas than in rural areas in China^2,3^, e.g., more than 20% of children and adolescents in Beijing, the capital of China and one of the world’s largest cities with a population of 21.7 million in 2015, were overweight or obese^4^.

The prevalence of hypertension among children has also increased in China^5–8^. Elevated blood pressure (BP) in children has become an important public health issue. A national study reported that the prevalence of hypertension among children aged 6–17 years increased from 7.1% in 1991 to 14.6% in 2004^6^. A recent study reported that 20.2% of boys and 16.3% of girls aged 6–14 years had elevated BP, and it was found to be common in obese children^7^. A study based on data from the China Health and Nutrition Survey (1991–2011) showed that the prevalence of hypertension among children increased over the past two decades regardless of sex or geographic area, and it was consistent with the upward trend of the prevalence of obesity^8^.

Studies have suggested that general and central obesity are predisposing factors for hypertension^9,10^. Higher body mass index (BMI) during childhood has been associated with an increased risk of fatal and nonfatal cardiovascular events during adulthood^11^. Adults with excess body fat accumulation in the early stages of life are more likely to have cardiovascular and metabolic diseases^12^. Children whose overweight and obesity persist into adulthood have an increased risk of having hypertension, type 2 diabetes, dyslipidemia, and carotid artery atherosclerosis than those who were never afflicted with overweight and obesity^13^.

Findings of previous studies are mixed regarding the associations of adiposity measurements with elevated BP in children, and knowledge about children in countries like China under rapid social transitions is limited. Some studies have suggested BMI and obesity were strong predictors of childhood elevated BP^14–16^, some reported that waist circumference (WC) and waist-to-height ratio (WHtR) are more strongly associated with elevated BP than BMI^17,18^, and others showed that these measurements had a similar association with elevated BP^19,20^. While there is a large sex difference in obesity rates in China^1,2^, few studies have examined these sex differences in the association between BMI (and obesity) and BP (and hypertension). The findings for sex-specific differences associated with obesity and hypertension can help direct different screening programs in adolescent boys and girls for hypertension, and target specific weight-loss initiatives for boys and girls^21^.

Over the past two decades, China has seen a marked growth of childhood overweight/obesity^1,3^, which is attributed to change of lifestyle (e.g., increased sedentary time due to motorized transportation, computer, internet, etc. and increased access to Western-style food)^4,22^. The combined prevalence of childhood overweight and obesity in urban China was 30% and 16% for boys and girls, respectively, increasing by 100% over the past two decades^2,23^. Studying the prevalence of obesity and hypertension in mega-cities in China is critical for fighting the epidemic. This is because China is under rapid urbanization and most future population growth is expected to occur in urban areas^24^.

To address the research gaps, this study examined the prevalence of general obesity, central obesity, and hypertension using data collected from children in four mega-cities across China, and studied the associations of general and central obesity with hypertension and their related sex differences.

## Methods and Materials

### Study design and participants

Data were collected by local collaborators (from local CDC and universities) in four major cities across China, including Beijing (China’s capital, in North China), Shanghai (China’s largest city, located in Southeast China), Nanjing (China’s old capital before 1949, in Southeast China), and Xi’an (in Northwest China) in October 2015. We contacted school administrators first to get their approval, and approached students through class teachers and recruited students and their parents after obtaining their assent and consent. Modest incentives like office supplies were provided to research subjects. The data were from 1648 students (aged 7–16 years) plus their parents, and related school administrators, physical education teachers, and/or school nurses from 16 schools (8 primary schools and 8 middle schools). The data contain rich information such as child growth and health, family characteristics, home, community and school environment, dietary intake, physical activity, and social network. The present analysis included 1626 students with complete data.

This study was conducted according to the guidelines laid down in the Declaration of Helsinki. All procedures involving human subjects were approved by the Ethical Committee of The State University of New York at Buffalo and related collaborative institutes in China. Written informed consent was obtained from all subjects.

### Key study variables

Anthropometric measures and BP data were obtained through physical examination conducted by personnel (physicians or nurses) trained by our research team using standardized protocols and calibrated equipment. Data collection was administered in the enrolled elementary and middle schools.

#### Outcome variable: hypertension

BP was measured twice using Omron HBP-1300 professional BP monitor. The second measure was recorded if the difference between two measures was less than 10 mm Hg. A third measure was performed if the difference of the first two measures was more than 10 mm Hg, and any two measures with a difference less than 10 mm Hg were recorded. Based on national data, BP reference standards for Chinese children and adolescents were established in 2010^25^, and the reference values of systolic blood pressure (SBP) and diastolic blood pressure (DBP) percentiles for Chinese children and adolescents were applied in this study, i.e., hypertension was defined as SBP and/or DBP ≥ 95^th^ percentile for age and gender.

#### Independent variables: general obesity, central obesity

Height was measured by a Seca 213 Portable Stadiometer Height-Rod with a precision of 0.1 centimeter; body weight was measured by a Seca 877 electronic flat scale with a precision of 0.1 kilogram; and WC was measured by a tape with a precision of 0.1 centimeter. BMI was calculated as weight in kilograms divided by height in meters squared. Overweight and obesity were determined according to the Working Group on Obesity in China (WGOC) age- and gender**-**specific BMI cutoffs points, which were developed based on age-sex-specific BMI curves that correspond to BMI cutoffs of 24 and 28 for overweight and obesity, respectively, for adults in China^26^. WHtR ≥ 0.48 was used to define central obesity for Chinese children^27^.

#### Covariates

Child factors included school level (primary school, middle school), sex, age, and location (Beijing, Shanghai, Nanjing, and Xi’an). Parental education level was categorized as “elementary school or below,” “middle, high, or vocational schools,” and “college or above.”

### Statistical analysis

First, characteristics of participants were described: for continuous variables, presented as means (standard deviation, SD); for categorical variables, as counts and percentages. Chi-square test or t-test was used to examine whether there was a difference by characteristics such as child sex and type of school. Then, correlations between BMI, WC, WHtR, SBP, and DBP with their level of significance were examined. Third, mixed models were used to examine associations between BP and BMI, WC, and WHtR for all, stratified by sex and by region, after adjusting for covariates. Finally, mixed models were used to examine the adjusted associations of general and central obesity with hypertension for all, by sex and by region.

With regard to using mixed models, the initial model included BP (i.e., SBP or DBP) and hypertension as the dependent variables. School was included in the model as a random effect (intercept). Including school as a random effect in the model resulted in a smaller Bayesian information criterion, indicating an improved model. All descriptive, univariate, and regression analyses were performed in SAS version 9.4 (SAS Institute, Cary, NC, USA). A significance level of α = 0.05 at two sides was used.

## Results

Table 1 shows the characteristics of the children. Overall, 11.1% had general obesity, 19.7% had central obesity, and 9.0% had hypertension. More boys than girls had general and central obesity (15.2% vs. 6.9%; 27.4% vs. 11.7%, respectively, both P < 0.0001). Boys had higher SBP than girls (107.2 [12.4] mm Hg vs. 104.8 [11.6] mm Hg, P < 0.0001), but had lower DBP (60.4 [7.7] mm Hg vs. 61.4 [8.1] mm Hg, P = 0.0166). There was no statistically significant difference in the prevalence of hypertension between boys and girls (9.3% vs. 8.8%, P = 0.7341).

**Table 1:** Characteristics of Chinese children aged 7–16 years included in the Childhood Obesity Study in China Mega-cities 2015 survey.

	Overall (n = 1 626)	Boys (n = 830)	Girls (n = 796)	P value^*^
**Child outcomes**				
*Obesity-related variables*				
BMI, mean (SD)	19.2 (3.9)	19.7 (4.0)	18.6 (3.8)	<0.0001
Waist circumference, mean (SD)	66.0 (10.3)	68.2 (11.1)	63.7 (9.0)	<0.0001
WHtR, mean (SD)	0.44 (0.06)	0.45 (0.06)	0.42 (0.05)	<0.0001
General obesity (yes), n (%)	180 (11.1)	126 (15.2)	55 (6.9)	<0.0001
Central obesity (yes), n (%)	320 (19.7)	227 (27.4)	93 (11.7)	<0.0001
*Blood pressure*				
SBP, mean (SD)	106.0 (12.1)	107.2 (12.4)	104.8 (11.6)	<0.0001
DBP, mean (SD)	60.9 (7.9)	60.4 (7.7)	61.4 (8.1)	0.0166
Hypertension (yes), n (%)	146 (9.0)	77(9.3)	70 (8.8)	0.7341
**Child factors**				
*Age (in years), mean (SD)*	11.6 (2.0)	11.6 (2.0)	11.5 (2.1)	0.9171
*Location, n (%)*				0.6176
Beijing	439 (27.0)	213 (25.7)	226 (28.4)	
Shanghai	400 (24.6)	212 (25.5)	188 (23.6)	
Nanjing	396 (24.4)	204 (24.6)	192 (24.1)	
Xi’an	391 (24.1)	201 (24.2)	190 (23.9)	
*School level, n (%)*				0.7453
Primary school	839 (51.6)	425 (51.2)	414 (52.0)	
Middle school	787 (48.4)	405 (48.8)	382 (48.0)	
**Maternal factors**				
*BMI, mean (SD)*	22.1 (3.2)	21.9 (2.8)	22.4 (3.4)	0.0007
*Education level, n (%)*				<0.0001
Elementary school or below	402 (25.6)	242 (30.5)	160 (20.7)	
Middle, high, or vocational schools	452 (28.8)	215 (27.1)	237 (30.6)	
College or above	715 (45.6)	337 (42.4)	378 (48.8)	

BMI, body mass index; SD, standard deviation; WHtR, waist-height ratio; SBP, systolic blood pressure; DBP, diastolic blood pressure.

*P value was calculated from either chi-squared test or t-test.

Correlations between BMI, WC, WHtR, and BP with level of significance are shown in Table 2. There were significantly (P < 0.001) positive correlations. The relationship of BMI, WC, and WHtR with SBP (*r* = 0.43, 0.44, and 0.35, respectively, all P < 0.001) was stronger than that for DBP (*r* = 0.19, 0.19, and 0.15, respectively, all P < 0.001). There was also a highly positive correlation between BMI, WC, and WHtR (*r* = 0.88 for BMI and WC, *r* = 0.87 for BMI and WHtR, and *r* = 0.94 for WC and WHtR, all P < 0.001).

**Table 2:** Correlation matrix between body mass index, waist circumference, waist-to-height ratio, and blood pressure among Chinese children aged 7–16 years: The Childhood Obesity Study in China Mega-cities (n = 1626)^†^.

	BMI	WC	WHtR	SBP	DBP
(1) BMI	1				
(2) WC	0.88^***^	1			
(3) WHtR	0.87^***^	0.94^***^	1		
(4) SBP	0.43^***^	0.44^***^	0.35^***^	1	
(5) DBP	0.19^***^	0.19^***^	0.15^***^	0.58^***^	1

BMI, body mass index; WC, waist circumference; WHtR, waist-to-height ratio; SBP, systolic blood pressure; DBP, diastolic blood pressure.

^†^Adjusted for age and city.

^***^Correlation was significant at P < 0.001 level (2-tailed).

Mixed models showed a positive association between BP (SBP and DBP) and BMI, WC, and WHtR, regardless of sex and region (see Table 3). The models adjusted for sex, age, city, and mother’s education. Table 4 shows the associations of general and central obesity with hypertension among all, by sex, and by region. Both general obesity (OR = 5.94, 95% confidence interval [CI]: 3.69–9.55) and central obesity (OR = 3.45, 95% CI: 2.27–5.23) were positively associated with hypertension.

**Table 3:** Mixed models for associations between adiposity measures and blood pressure among Chinese children, stratified by sex and by region: The Childhood Obesity Study in China Mega-cities (n = 1626).

	BMI			WC			WHtR		
	Beta	SE	P value	Beta	SE	P value	Beta	SE	P value
**(A) For SBP**									
All^†^	1.22	0.06	<0.0001	0.50	0.03	<0.0001	65.08	4.53	<0.0001
Sex									
Boys^‡^	1.32	0.09	<0.0001	0.49	0.03	<0.0001	64.67	5.82	<0.0001
Girls^‡^	1.07	0.10	<0.0001	0.50	0.04	<0.0001	67.23	7.21	<0.0001
Region									
Beijing^§^	1.41	0.13	<0.0001	0.55	0.05	<0.0001	70.80	9.07	<0.0001
Shanghai^§^	0.80	0.12	<0.0001	0.33	0.04	<0.0001	45.21	7.71	<0.0001
Nanjing^§^	1.16	0.13	<0.0001	0.52	0.06	<0.0001	69.40	9.46	<0.0001
Xi’an^§^	1.47	0.14	<0.0001	0.62	0.06	<0.0001	78.55	10.06	<0.0001
**(B) For DBP**									
All^†^	0.43	0.05	<0.0001	0.18	0.02	<0.0001	23.52	3.27	<0.0001
Sex									
Boys^‡^	0.46	0.06	<0.0001	0.17	0.02	<0.0001	22.68	4.04	<0.0001
Girls^‡^	0.39	0.07	<0.0001	0.18	0.03	<0.0001	24.48	5.49	<0.0001
Region									
Beijing^§^	0.39	0.09	<0.0001	0.13	0.04	0.0004	19.09	6.11	0.0019
Shanghai^§^	0.63	0.09	<0.0001	0.26	0.03	<0.0001	36.28	5.68	<0.0001
Nanjing^§^	0.36	0.10	0.0002	0.15	0.04	0.0004	22.34	6.98	0.0015
Xi’an^§^	0.38	0.11	0.0006	0.16	0.05	0.0006	15.84	7.58	0.0373

BMI, body mass index; WC, waist circumference; WHtR, waist-to-height ratio; SE, standard error; SBP, systolic blood pressure; DBP, diastolic blood pressure.

Linear regression models were fit for each outcome and stratified by sex and region.

^†^Adjusted for sex, age, city, and mother’s education.

^‡^Adjusted for age, city, and mother’s education.

^§^Adjusted for age, sex, and mother’s education.

**Table 4:** Mixed models for associations between general and central obesity and hypertension among Chinese children, and stratified by gender and by region: The Childhood Obesity Study in China Mega-cities (n = 1626).

	General obesity			Central obesity		
	OR	95% CI	P value	OR	95% CI	P value
All^†^	5.94	3.69–9.55	<0.0001	3.45	2.27–5.23	<0.0001
Sex						
Boys^‡^	5.65	3.05–10.48	<0.0001	3.17	1.81–5.55	0.0005
Girls^‡^	6.14	2.87–13.16	0.0001	3.85	1.99–7.46	0.0006
Regions						
Beijing^§^	5.07	1.29–19.90	0.0324	4.13	1.22–14.00	0.0344
Shanghai^§^	2.85	0.54–15.08	0.1394	1.30	0.28–6.02	0.6247
Nanjing^§^	5.65	1.36–23.55	0.0306	4.33	1.32–14.21	0.0294
Xi’an^§^	16.53	3.61–75.65	0.0099	5.86	1.45–23.72	0.0275

Abbreviations: OR, odds ratio; CI, confidence interval. General obesity was defined based on body mass index; central obesity, based on wait circumference.

^†^Adjusted for sex, age, city, and mother’s education.

^‡^Adjusted for age, city, and mother’s education.

^§^Adjusted for age, sex, and mother’s education.

## Discussion

Hypertension is the leading risk factor for cardiovascular disease mortality and is responsible for 13% of deaths globally^28^. Studies have suggested that childhood BP was associated with BP in adulthood and that children with high BP had a higher probability of developing hypertension as adolescence and adults than children with low BP^29,30^. Childhood hypertension has become an important public health issue in China. The present study found that about 9% of children in mega-cities in China had hypertension, 11.1% were obese, and 19.7% were centrally obese. The prevalence of general obesity and central obesity in boys more than doubled those in girls (15.2% vs. 6.9%; 27.4% vs. 11.7%, respectively, both P < 0.0001). These rates were higher than the reported national average. A national study based on the Chinese National Surveys on Students’ Constitution and Health data reported the national prevalence of obesity was 8.1% (10.1% in urban areas, 6.4% in rural areas, 10.9% in boys, and 5.1% in girls) among children in 2010^2^.

Interventions to prevent childhood obesity and hypertension are urgently needed in China. The prevalence of hypertension was 9.0% in our studied children and is similar to that in American children (9.6% in 2009–2012)^31^. Of more concern, a 2010 nationwide cross-sectional study reported an even higher prevalence of hypertension than ours: 20.2% of Chinese boys and 16.3% of girls had elevated BP^7^. The China Health and Nutrition Survey data reported that hypertension prevalence among children in 2009 was 13.8% (16.2% in urban areas and 13.0% in rural areas)^32^. The higher national rates than those in our study could be due to the difference in children’s diet in different regions, e.g., people in rural areas in China had higher salt intake than those in urban areas^33^. Moreover, in China, people in urban areas, especially in mega-cities, have higher socioeconomic status than their counterparts in rural areas. A study showed that children with low socioeconomic status were more likely to have elevated BP levels^34^. The prevalence of hypertension among children in rural areas of China may be higher than the prevalence (9.0%) among children in the four mega-cities reported by our study. More research is needed in the future to understand the underlying reasons for these differences.

While the prevalence of hypertension in U.S. children and adolescents has declined during the past decade, the prevalence in Chinese children has been increasing^31^. The China Health and Nutrition Survey data showed that hypertension prevalence among children 6–17 years increased from 7.6% in 1993 to 13.8% in 2009^32^, while the prevalence among children aged 8–17 years increased from 6.9% in 1991 to 10.7% in 2011(based on U.S. reference)^8^. The increasing trend appeared regardless of sex, area, and definition of hypertension used (recommended by U.S. or Chinese working groups) and was consistent with the increase of obesity^8,32^. At least part of the increase of hypertension prevalence among Chinese children is due to the increased obesity prevalence.

Consistent with results among U.S. and European children^35,36^, our study showed that among Chinese children, SBP and DBP were positively associated with BMI and WC, and that general and central obesity were positively associated with childhood hypertension regardless of sex and region. Generally obese children were 5.94 times and centrally obese children were 3.45 times more likely to have hypertension than their normal-weight counterparts. Although the pathogenesis of obesity-related hypertension, e.g. insulin resistance, sympathetic nervous system stimulation, renin-angiotensin-aldosterone system activation, increased secretion of leptin and other bioactive factors, were demonstrated in previous studies, the mechanisms underlying obesity-associated hypertension and other associated metabolic disease remain unclear and need more studies^9,37^.

Nevertheless, effective programs and policies to promote healthy growth and household nutrition security and to protect children from inducements to be inactive or to overconsume foods of poor nutritional quality are needed at global, national, and regional levels to tackle childhood obesity and hypertension^38–40^. A broad package of measures to prevent childhood obesity is needed and it is unlikely that a focus on any one component or on the health sector alone could be effective^41^. Schools offer a key opportunity for programmatic action, such as standards for school meals, elimination of unhealthy foods (e.g., sugar-sweetened beverages and energy-dense, nutrient-poor foods) in the school environment, informed nutrition education as part of the core curriculum, and ensuring levels of physical activity for all children according to WHO recommendations^40^. Our previous study evidenced the important role of school in the prevention of childhood obesity: pocket money is a risk factor for unhealthy eating and obesity among Chinese children, while school policies on unhealthy food restrictions may buffer this negative influence^42^.

Overall, our findings on the association between obesity and hypertension are consistent with those in another sample of Chinese children^19^ and in German children^20^, but are inconsistent with studies which suggest that central obesity indicators (e.g., WC, WHtR) are better than a general obesity indicator (i.e., BMI) to predict hypertension. For example, a study among Asian Indian girls reported that WC is the best adiposity measure in predicting hypertension, but there was no significant association of BMI and WHtR with hypertension^17^.

Our study found that boys had a higher mean SBP than girls (107.2 mm Hg vs. 104.8 mm Hg, P < 0.0001) and a slightly higher prevalence of hypertension than girls (9.3% vs. 8.8%, P = 0.7341); however, boys’ mean DBP was lower than girls (60.4 mm Hg vs. 61.4 mm Hg, P = 0.0166). This result is consistent with previous studies to the effect that elevated SBP occurs more frequently in males than females and boys are more likely than girls to develop high SBP as they approach adulthood^43^.

Despite its important strengths, this study has some limitations. First, the cross-sectional design did not test for a causal association between obesity and hypertension. Second, the diagnosis of hypertension is based on the BP measurement at a single visit on the examination day, and not as recommended for clinical practice, that is the measurement must be repeated on at least 2 additional occasions to confirm the diagnosis. It has been reported that BP measurement at a single visit usually overestimates hypertension prevalence^44^. Nevertheless, BP measurement at a single visit is often used in epidemiologic studies. Third, comparison of hypertension prevalence across studies needs to be cautious, even among studies conducted in China, due to differences in BP measurement devices and hypertension classifications being used. For example, the study of Zhai *et al*.^7^ used age-specific and height-specific references recommended by the U.S. National High Blood Pressure Education Program Working Group on High Blood Pressure in Children and Adolescents, while the study by Xi *et al*.^32^ used the Chinese age-sex-specific reference as we did in this study, but they used a mercurial sphygmomanometer to measure BP, which is different from our study using an Omron HBP-1300 professional blood pressure monitor. Differences in BP measurements and the study populations likely have contributed to different findings across studies.

In conclusion, the prevalence of general obesity, central obesity, and hypertension was high among children in these four mega-cities across China. Their BMI, WC, and WHtR were positively associated with SBP and DBP. Children with general obesity and central obesity were more likely to have hypertension. Boys had a higher mean SBP than girls. Effective strategies are urgently needed to prevent childhood obesity and hypertension in China.
